# Sarcopenic Obesity in Individuals With Neurodisabilities: The SarcObeNDS Study

**DOI:** 10.3389/fendo.2022.868298

**Published:** 2022-07-19

**Authors:** Yannis Dionyssiotis, Konstantinos Prokopidis, George Trovas, Maria-Christina Papadatou, Nikolaos Ananidis, Vasileios Tragoulias, Eleni Lazarou, Evangelia Christaki, Marilena Domazou, Antonios Galanos, Minos Tyllianakis

**Affiliations:** ^1^ Spinal Cord Injury Rehabilitation Clinic, Patras University Hospital, Patras, Greece; ^2^ Laboratory for Research of the Musculoskeletal System, National and Kapodistrian University of Athens, Kifissia, Greece; ^3^ 1st Physical Medicine and Rehabilitation Department, National Rehabilitation Center EKA, Athens, Greece; ^4^ Department of Musculoskeletal Biology, Institute of Life Course and Medical Sciences, University of Liverpool, Liverpool, United Kingdom; ^5^ Radiology Department, National Rehabilitation Center EKA, Athens, Greece

**Keywords:** sarcopenic obesity, sarcopenia, spinal cord injury, stroke, traumatic brain injury, skeletal mass index

## Abstract

**Introduction:**

Patients with neurodisabilities (NDS) are prone to alterations in body composition. Sarcopenic obesity (SO) is a condition characterized by increased adipose tissue accompanied by sarcopenia. The aim of this study was to investigate the prevalence of SO in patients with NDS, including stroke, spinal cord, and traumatic brain injuries.

**Methods:**

The study Sarcopenic Obesity in NeuroDisabled Subjects (acronym: SarcObeNDS) was a cross-sectional study of hospitalized patients (*n* = 82) and healthy controls (*n* = 32) with a mean age of 60.00 ± 14.22 years old. SO and sarcopenia were assessed through total body fat % (TBF %), fat mass index (fat mass to height^2^: FMI = FM/h^2^; kg/m^2^), and skeletal muscle index (appendicular skeletal muscle to height^2^: SMI = ASM/h^2^; kg/m^2^) *via* full-body dual-energy X-ray absorptiometry (DXA). This study was registered in the international database ClinicalTrials.gov with the unique identification number NCT03863379.

**Results:**

A statistically significant difference was found in SMI (7.18 ± 0.95 vs. 6.00 ± 1.13 kg/m^2^, *p* < 0.001) between controls and patients with NDS. No statistical significance was found for TBF (*p* = 0.783) and FMI (*p* = 0.143) between groups. The results remained the same after controlling the results for gender and BMI. A strong positive correlation was demonstrated between BMI and TBF for the total population (*r* = 0.616, *p* < 0.001), the control group (*r* = 0.616, *p* < 0.001), and patients with NDS (*r* = 0.728, *p* < 0.001).

**Conclusion:**

In summary, we observed significantly lower BMI and SMI scores in both genders compared to healthy controls. At the clinical level, a timely diagnosis and rapid treatment of sarcopenia and/or obesity in this population may prevent further metabolic repercussions accompanied by higher functional decline and lower quality of life.

## Introduction

Individuals with neurodisabilities (NDS) are prone to immobilization which may contribute to changes in body composition. The potential risks involved following such alterations include loss of lean mass (LM) and bone mineral density (BMD), as well as higher fat mass (FM) (1). Accelerating age-related skeletal muscle perturbations leading to muscle mass and functional decline (2) are known as sarcopenia (3), which may be highly prevalent in patients with NDS who are characterized by limited physical and cognitive function (4). Additionally, obesity is another major risk factor for metabolic and cardiovascular morbidity and increased mortality. Prolonged physical inactivity and sedentarism may predispose individuals with NDS to a greater risk of both sarcopenia and obesity (5). More importantly, patients with NDS are at an alarming risk of depression and reduced mobility and quality of life (6, 7), which may be potentiated by reductions in skeletal muscle mass and physical performance (8). Therefore, it is imperative to assess the occurring changes in body composition in individuals with NDS, aiming to prevent the accruing catabolic and psychological impact of sarcopenia and/or obesity.

Moreover, the coexistence of sarcopenia and obesity has merged a new entity, defined as sarcopenic obesity (SO) (9). This term is currently based on body mass index (BMI) (>25 kg/m^2^ for overweight and >30 kg/m^2^ for people with obesity) or waist circumference (WC) measurements (>102 cm in men; >88 cm in women), which may not reliably correspond with levels of adiposity (10, 11). According to the European Working Group on Sarcopenia in Older People 2 (EWGOSP2), a component of sarcopenia is defined by a skeletal muscle index (SMI) of <7.0 kg/m^2^ (men) and <5.5 kg/m^2^ (women) (3), whereas individuals with a whole-body fat of >28% (men) and >40% (women) aged above 40 years are considered to have obesity (12). Individuals with sarcopenia and obesity are more susceptible to metabolic disorders than cohorts exhibiting a phenotype that involves sarcopenia or obesity alone (13). The complex interplay of common pathophysiological mechanisms such as increased proinflammatory cytokines, oxidative stress, insulin resistance, hormonal perturbations, and decreased physical activity underlies the close relationship between sarcopenia and obesity (14). Hence, this vicious cycle may amplify a concomitant accumulation of adipose tissue and loss of skeletal muscle mass. Therefore, investigating the prevalence of SO in populations with NDS may provide valuable insights that would enable targeted interventions aiming to counteract both sarcopenia and obesity. The aim of this study was to investigate the prevalence of the components of SO in patients with NDS. We hypothesized that patients with NDS would exhibit lower overall muscle mass and increased fat mass compared to healthy individuals.

## Methods

### Demographics

Patients with NDS, including stroke, spinal cord injury, and traumatic brain injury (*n* = 82), aged 60.00 ± 14.22 years old in the subacute phase (3–6 months post-injury) and hospitalized during the time of examination were included in this study. The control group (*n* = 32) consisted of healthy volunteers working in the laboratory and hospital.

**Figure 1:**
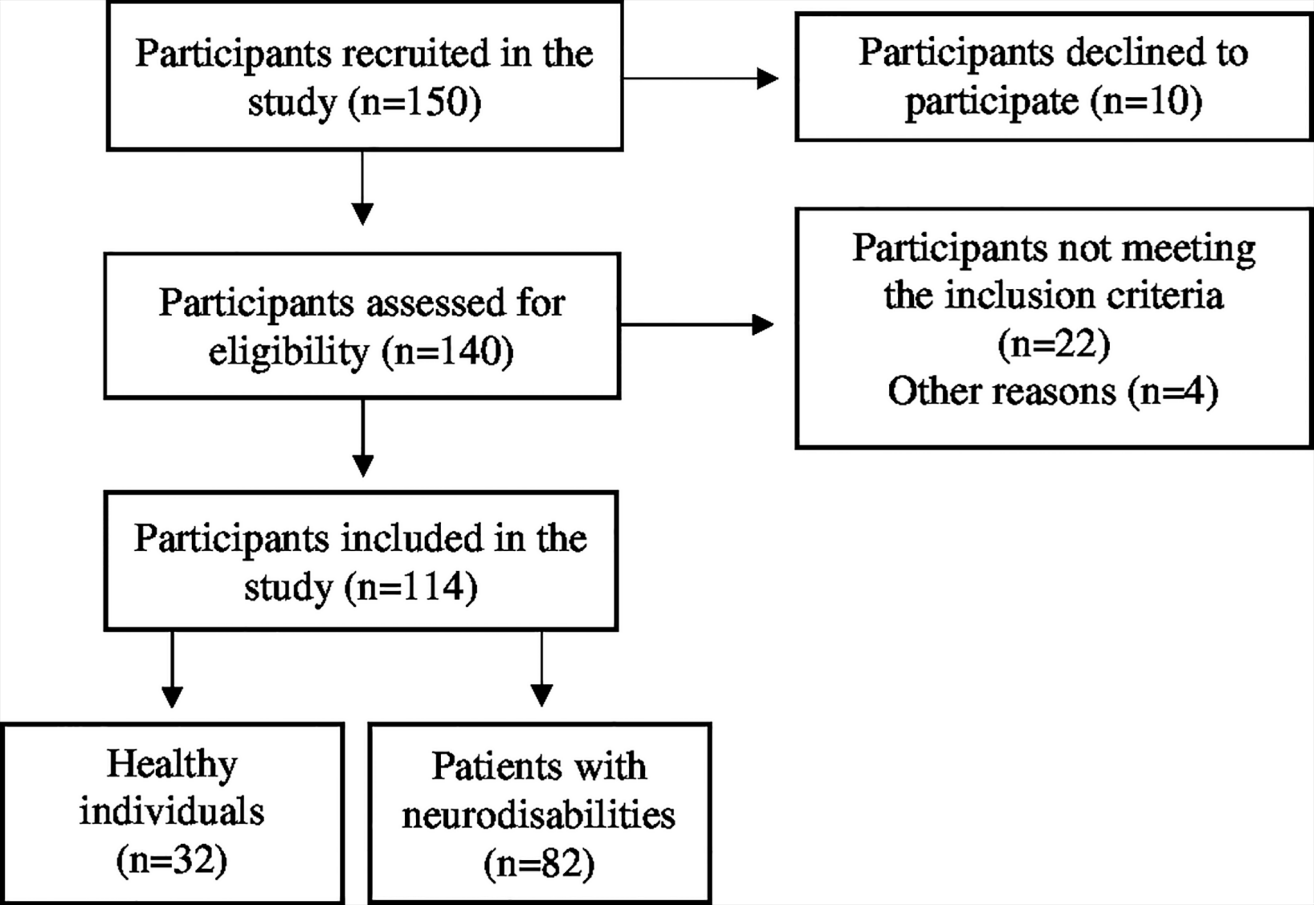
Flowchart of the screening process for the selection of included participants.

Participants were included if they had sustained a recent (below 3 months) neurodisability, were medically stable, could provide informed consent, and had medical clearance to participate in the study. Exclusion criteria were being under 18 years of age; having an American Spinal Injury Association Impairment Scale (AIS) D (50% of the muscles below the injury level are scored as 3 or above) at baseline [only for subjects with spinal cord injury (SCI)]; suffering from metabolic bone disease, including lytic or renal bone disease, or senile osteoporosis; having prior exposure to drugs that affect bone metabolism [amino-bisphosphonate, high-dose glucocorticoids, cyclosporine, anti-epileptic drugs (AEDs)]; and finally, having a cardiac pacemaker or known history of epilepsy (**Figure 1**).

Anthropometric factors such as age, height, weight, and BMI were recorded in all subjects. In patients with NDS, height was measured in a supine position before the examination, while the height of the control group was measured using a wall-mounted ruler in a standing position. The body weight of the controls was measured on a standard weight scale, while the body weight of the participants with NDS was assessed in a sitting position on a wheelchair following subtraction of the wheelchair’s weight. BMI was calculated for each subject (BMI = weight/height^2^; kg/m^2^). Controls were considered healthy after physical examination and comprehensive medical history review, which was free of any previous fracture, endocrine or metabolic bone disease, cancer, drug abuse, alcoholism, NDS, and hepatic or renal disorders.

This study was conducted at the 1st Physical Medicine and Rehabilitation Department of the National Rehabilitation Center EKA (Ethniko Kentro Apokatastasis) in Athens, in cooperation with the Radiology Department of the National Rehabilitation Center EKA and the Laboratory for Research of the Musculoskeletal System at the University of Athens in KAT Hospital, Kifissia in Greece. The protocol was designed according to the Declaration of Helsinki and approved by the Ethics Committee of the University of Athens. All subjects provided written informed consent in order to take part in this study. The study was registered in the international database ClinicalTrials.gov with the unique identification number NCT03863379.

### Measurements

All subjects were examined using a whole-body dual-energy X-ray absorptiometry (DXA) scan (Lunar, USA) to estimate the regional, total body, and percentage of LM and FM (g). SO and sarcopenia were assessed using the following indicators: total body fat % (TBF %), fat mass index to height (h^2^) (kg/m^2^)—FMI = FM/h^2^, and skeletal muscle index [ASM to height (h)^2^ (kg/m^2^) − SMI = ASM/h^2^]. Reference values recommended by the European Working Group of Sarcopenia in Older People 2 (EWGOSP2) (SMI < 7.0 and <5.5 kg/m^2^) were used to indicate a domain of sarcopenia (3).

### Statistical Analysis

Quantitative and qualitative variables are represented by the mean and standard deviation (mean ± SD) or the mean value and standard error (mean ± SE) for two-way ANOVA model analysis and frequencies (*n*) and percentages (%), respectively. The normal distributions of quantitative variables were examined using the Kolmogorov–Smirnov test. The quantitative and qualitative demographic characteristics as well as the markers of sarcopenia between the patients with DNS and the control group were compared using the independent samples *t*-test and the Fisher’s exact test, respectively. The assumptions of normality and homogeneity of variance were also examined. The correlation of BMI with TBF was estimated using the Pearson correlation coefficient due to the normality of the variables. Comparison between correlation coefficients was performed using the Fisher r-to-z transformation method. The two-way ANOVA model using “gender” (between groups) and “disabled status, NDS” (between groups) as factors was used to examine the influence of gender on differences in sarcopenia markers between compared groups. The same model was used adding the BMI variable as a covariate to examine the differences of sarcopenia markers between compared groups adjusted for BMI differences. All statistical analyses were performed using the statistical package SPSS version 21.0 (IBM Corporation, Somers, NY, USA). A *p*-value of <0.05 was set as the level of statistical significance.

## Results

For both men and women, a statistically significant difference in terms of BMI between the control group and patients with NDS was observed (*p* = 0.012); however, no significant differences were found concerning age (*p* = 0.392), gender (*p* = 0.208), height (*p* = 0.216), and weight (*p* = 0.192) (**Table 1**).

**Table 1:** Comparison of the demographic characteristics of patients with NDS and the control group.

	Total (114)	Control (32)	Disabled (82)	*p*-value
Age (years)^a^	60.00 ± 14.22	58.16 ± 17.39	60.71 ± 12.84	0.392
Height (m)^a^	1.66 ± 0.11	1.64 ± 0.13	1.67 ± 0.10	0.216
Weight (kg)^a^	68.73 ± 13.55	71.39 ± 11.86	67.69 ± 14.09	0.192
BMI (kg/m^2^)^a^	24.95 ± 4.66	26.70 ± 4.54	24.27 ± 4.55	**0.012**
Gender^b^: male/female (%)	65 (57%)/49 (43%)	15 (47%)/17 (53%)	50 (61%)/32 (39%)	0.208

All variables are presented as mean ± SD, except gender which is presented as frequencies (percentage).

a Independent samples t-test.

b Fisher’s exact test.

For men, a statistically significant difference in terms of BMI between the control group and patients with NDS was observed (*p* = 0.035); however, no significant differences were found concerning age (*p* = 0.571), height (*p* = 0.879), and weight (*p* = 0.075). For women, no significant differences between the control group and patients with NDS were found concerning age (*p* = 0.577), height (*p* = 0.449), BMI (*p* = 0.224), and weight (*p* = 0.606) (**Table 2**).

**Table 2:** Comparison of the baseline demographic characteristics of patients with NDS and the control group according to gender.

		Control (32)	Disabled (82)	*p*-value
Male	Age (years)^a^	58.73 ± 16.17	61.00 ± 12.65	0.571
	Height (m)^a^	1.73 ± 0.10	1.72 ± 0.09	0.879
	Weight (kg)^a^	78.27 ± 10.75	70.42 ± 14.01	0.075
	BMI (kg/m^2^)^a^	26.45 ± 4.20	23.75 ± 4.27	**0.035**
Female	Age (years)^a^	57.65 ± 18.88	60.25 ± 13.30	0.577
	Height (m)^a^	1.57 ± 0.10	1.59 ± 0.06	0.449
	Weight (kg)^a^	65.32 ± 9.36	63.44 ± 13.31	0.606
	BMI (kg/m^2^)^a^	26.92 ± 4.93	25.10 ± 4.91	0.224

All variables are presented as mean ± SD.

a Independent samples t-test.

A statistically significant difference between the control group and patients with NDS was also found concerning SMI (*p* < 0.001), although there was no difference between TBF (*p* = 0.793) and FMI (*p* = 0.143) (**Table 3**).

**Table 3:** Comparison of clinical indices between patients with NDS and the control group.

	Control (32)	Disabled (82)	Mean difference (95% CI)	*p*-value
TBF (%)^a^	35.21 ± 10.35	35.92 ± 12.95	−0.71 (−5.78/4.37)	0.783
SMI (kg/m²)^a^	7.18 ± 0.95	6.00 ± 1.13	1.18 (0.73/1.63)	**<0.001**
FMI (kg/m²)^a^	9.73 ± 3.86	8.49 ± 4.11	1.24 (−0.43/2.91)	0.143

All variables are presented as mean ± standard deviation (SD).

FMI, fat mass index; SMI, skeletal muscle mass index; TBF, total body fat.

a Independent samples t-test.

Using the two-way analysis of variance model, we examined the influence of gender in relation to disability status. There was no statistically significant interaction between gender and disability status (control vs. NDS subjects) for TBF (*p* = 0.889), SMI (*p* = 0.832), and FMI (*p* = 0.511). This significance remained unchanged between controls and patients with NDS in both men (*p* < 0.001) and women (*p* < 0.001) for the SMI variable. No statistically significant difference between controls and individuals with NDS for TBF and FMI in neither men nor women [(*p* = 0.422 vs. *p* = 0.326) and (*p* = 0.248 vs. *p* = 0.830), respectively] was observed (**Table 4**).

**Table 4:** Two-way ANOVA for clinical indices using the factors “NDS” and “gender”.

	Male (65)		Mean difference (95% CI)	*p*-value_male_	Female (49)		Mean difference (95% CI)	*p*-value_female_	*p*-value_interaction_
	Control (15)	Disabled (50)			Control (17)	Disabled (32)			
TBF (%)	27.91 ± 2.62	30.32 ± 1.44	−2.4 (−8.3/3.5)	0.422	41.65 ± 2.49	44.67 ± 1.80	−3.0 (−9.1/3.00)	0.326	0.889
SMI (kg/m²)	7.78 ± 0.25	6.41 ± 0.14	1.4 (0.8/1.9)	**<0.001**	6.65 ± 0.23	5.35 ± 0.17	1.3 (0.7/1.9)	**<0.001**	0.832
FMI (kg/m²)	8.31 ± 0.95	7.05 ± 0.52	1.3 (−0.9/3.4)	0.248	10.99 ± 0.90	10.75 ± 0.66	0.2 (−2.5/2.0)	0.830	0.511

All variables are presented as mean ± SE (standard error); p-value_interaction_: interaction between “NDS” and “gender.” Residuals are normally distributed and there is homogeneity of variances based on Levene’s test.

FMI, fat mass index; SMI, skeletal muscle mass index; TBF, total body fat.

Using the two-way analysis of variance model, we examined the influence of gender in relation to disability status adjusted for the BMI measurement variable. There was no statistically significant interaction between gender and disability status (control vs. NDS subjects) for SMI (*p* = 0.990) and FMI (*p* = 0.623), which revealed that the difference between control and NDS subjects, regarding SMI and FMI indices, was not influenced by gender adjusted for BMI.

No statistically significant difference between controls and individuals with NDS for FMI in neither men nor women (*p* = 0.370 vs. *p* = 0.177, respectively) was observed. On the contrary, a statistically significant difference between controls and patients with NDS for the SMI was found in men and women (*p* < 0.001) (**Table 5**).

**Table 5:** Two-way ANOVA for clinical indices using the factors “NDS” and “gender” adjusted for BMI.

	Male (65)		Mean difference (95% CI)	*p*-value_male_	Female (49)		Mean difference (95% CI)	*p*-value_female_	*p*-value_interaction_
	Control (15)	Disabled (50)			Control (17)	Disabled (32)			
SMI (kg/m²)	7.63 ± 0.22	6.53 ± 0.12	1.1 (0.6/1.6)	**<0.001**	6.45 ± 0.21	5.34 ± 0.15	1.1 (0.6/1.6)	**<0.001**	0.990
FMI (kg/m²)	7.30 ± 0.55	7.87 ± 0.30	−0.6 (−1.8/0.7)	0.370	9.66 ± 0.51	10.65 ± 0.37	−1.0 (−2.2/0.3)	0.177	0.623

All variables are presented as mean ± SE adjusted for BMI; p-value_interaction_: interaction between “DNS” and “gender.” Residuals are normally distributed and there is homogeneity of variances based on Levene’s test.

FMI, fat mass index; SMI, skeletal muscle mass index.

A strong correlation between BMI and TBF in the total population (*r* = 0.673, *p* < 0.001), the control group (*r* = 0.616, *p* < 0.001), and patients with NDS (*r* = 0.728, *p* < 0.001) without differences in correlation coefficients between groups (*p* = 0.103) was displayed (**Table 6**).

**Table 6:** Correlation between total body fat (%) and BMI for patients with NDS, the control group control, and the total population.

	Pearson’s correlation coefficient	*p*-value
Total (114)	0.673	<0.001
Control (32)	0.616	<0.001
Disabled (82)	0.728	<0.001

## Discussion

This cross-sectional study explored the prevalence of indices of sarcopenic obesity in patients with NDS. Our findings revealed statistically significant associations between BMI and SMI in both genders. No statistical significance was found for TBF and FMI between groups. The results remained the same after controlling the results for gender and BMI. Eventually, a strong correlation between BMI and TBF in both the controls and patients with NDS was demonstrated, while no significant differences in TBF between both groups and genders were highlighted.

Previous studies have shown that the average BMI for individuals with SCI ranges between 21.7 and 28.9 kg/m^2^, independent of time since injury. This BMI range, however, fails to support the increased prevalence of obesity in people with SCI, considering a total body fat percentage ranging from 23% to as high as 40% in this population (15). During the initial stage of the injury, there are dramatic metabolic changes in patients with NDS leading to increased adiposity and protein catabolism, including increased energy expenditure and nitrogen excretion, and elevated catabolic, hormonal, and cytokine profiles in blood and tissue levels (16). Furthermore, another possible explanation highlighting lower values of BMI and potentially of lean body mass in men can be attributed to a higher risk of severe traffic accidents in comparison to women (17). However, the applicability of conventional BMI cutoff values has been widely questioned (18, 19). BMI is a sensitive marker that does not consider adipose and lean body mass tissue; thus, evaluating the rates of obesity by utilizing BMI scores in patients with NDS may be an imprecise tool in determining body weight status. Specifically, previous studies have shown that the current BMI cutoff points fail to identify most patients with SCI that had obesity, ending up using lower BMI cutoffs (>22 kg/m^2^) (18, 20). Individuals with identical BMI and/or TBF may have different body compositions; therefore, more accurate results may be derived by measuring FMI as opposed to either BMI or TBF (21). Hence, despite an association between BMI and FMI, the diagnostic accuracy of BMI in evaluating the prevalence of obesity is limited (22). Consequently, future clinical trials should utilize more efficient assessment tools in relation to body fat composition in patients with NDS.

Our findings unveiled a significant difference in SMI between controls and patients with NDS in both genders following adjustment for BMI. In the past, we utilized an SMI cutoff value of 5.8 kg/m^2^ in order to estimate the prevalence of sarcopenia in a cohort of individuals with chronic SCI (20). In that study, a large proportion of patients with SCI NDS were diagnosed with reduced muscle mass (~42%), reporting an overall mean SMI value of 6.4 kg/m^2^ for men and 5.35 kg/m^2^ for women. These findings are in line with research demonstrating that lean mass of the contralateral limb was lower compared to the ipsilateral limb after upper motor neuron injury in patients with stroke (23–25). Furthermore, in a prospective 1-year study, muscle mass was dramatically diminished in conjunction with an increase in body fat mass during the first 15 weeks of a lower limb injury, with total lean muscle mass losses of ~9.5% within 6 months and ~15% within a year after the injury (26). Eventually, decreased muscle mass and increased intramuscular fat have also been displayed in individuals with SCI (15). Elevated muscle atrophy is prominently observable during the subacute phase of NDS, particularly during the first 3 months of injury (27). Nevertheless, considering the accelerating muscle atrophy due to greater catabolic responses following injury and prolonged periods of physical inactivity, assessment of sarcopenia in patients with NDS may be a valuable prognostic tool that would enable practitioners and clinicians to optimize recovery of physical function and quality of life.

## Limitations

Our study was prone to several limitations. We did not perform sample size estimation due to difficulties in finding a sufficient control sample due to COVID-19. Specifically, in patients with NDS, the accuracy of skeletal muscle mass measured by DXA and the prevalence of obesity *via* BMI may be compromised due to existing limitations in both assessment tools (28, 29). More importantly, a lower ratio of muscle mass to adipose tissue indicates a lower proportion of muscle covering lean tissue mass. In this case, muscle mass may be overestimated by prediction models considering that skeletal muscle represents a certain proportion of fat-free mass (30). On the other hand, individuals who are underweight may be diagnosed with sarcopenia and/or low muscle mass despite a high muscle mass to body weight ratio. Hence, SMI could misclassify individuals with obesity by exhibiting an increase, while underweight populations may demonstrate a low SMI score.

## Conclusion

This cross-sectional study revealed an association between lower BMI and SMI scores in both genders compared to healthy controls. In individuals with NDS, optimizing body composition is imperative to reduce the risk of functional decline. At the clinical level, implementing accurate assessment tools to diagnose sarcopenia and obesity in this population may allow for timely treatments and prevent additional catabolic responses linked to reduced physical performance and quality of life.

## Data Availability Statement

The original contributions presented in the study are included in the article/supplementary material. Further inquiries can be directed to the corresponding author.

## Ethics Statement

The studies involving human participants were reviewed and approved by Ethics Committee of National Rehabilitation Center EKA, Athens, Greece. The patients/participants provided their written informed consent to participate in this study.

## Author Contributions

YD proposed the concept of the study, participated in sample collection, and wrote the manuscript. KP, GT, NA, MD, and MT edited the manuscript. M-CP and VT participated in sample collection. EL and EC were responsible for the DXA measurements. AG performed the statistical analysis. All authors contributed to the article and approved the submitted version.

## Conflict of Interest

The authors declare that the research was conducted in the absence of any commercial or financial relationships that could be construed as a potential conflict of interest.

## Publisher’s Note

All claims expressed in this article are solely those of the authors and do not necessarily represent those of their affiliated organizations, or those of the publisher, the editors and the reviewers. Any product that may be evaluated in this article, or claim that may be made by its manufacturer, is not guaranteed or endorsed by the publisher.
